# Population Size Estimation of Venue-Based Female Sex Workers in Ho Chi Minh City, Vietnam: Capture-Recapture Exercise

**DOI:** 10.2196/10906

**Published:** 2019-01-29

**Authors:** Giang Le, Nghia Khuu, Van Thi Thu Tieu, Phuc Duy Nguyen, Hoa Thi Yen Luong, Quang Duy Pham, Hau Phuc Tran, Thuong Vu Nguyen, Meade Morgan, Abu S Abdul-Quader

**Affiliations:** 1 Program Development Office United States Agency for International Development, Vietnam Hanoi Viet Nam; 2 Department for Disease Control and Prevention Pasteur Institute Ho Chi Minh City Viet Nam; 3 Ho Chi Minh City HIV/AIDS Center Ho Chi Minh City Viet Nam; 4 Division of Global HIV and Tuberculosis United States Centers for Disease Control and Prevention Hanoi Viet Nam; 5 Division of Global HIV and Tuberculosis United States Centers for Disease Control and Prevention Atlanta, GA United States

**Keywords:** population size estimation, venue-based, female sex workers, Ho Chi Minh City, capture-recapture

## Abstract

**Background:**

There is limited population size estimation of female sex workers (FSWs) in Ho Chi Minh City (HCMC)—the largest city in Vietnam. Only 1 population size estimation among venue-based female sex workers (VFSWs) was conducted in 2012 in HCMC. Appropriate estimates of the sizes of key populations are critical for resource allocation to prevent HIV infection.

**Objective:**

The aim of this study was to estimate the population size of the VFSWs from December 2016 to January 2017 in HCMC, Vietnam.

**Methods:**

A multistage capture-recapture study was conducted in HCMC. The capture procedures included selection of districts using stratified probability proportion to size, mapping to identify venues, approaching all VFSWs to screen their eligibility, and then distribution of a unique object (a small pink makeup bag) to all eligible VFSWs in all identified venues. The recapture exercise included equal probability random selection of a sample of venues from the initial mapping and then approaching FSWs in those venues to determine the number and proportion of women who received the unique object. The proportion and associated confidence bounds, calculated using sampling weights and accounting for study design, were then divided by the number of objects distributed to calculate the number of VFSWs in the selected districts. This was then multiplied by the inverse of the proportion of districts selected to calculate the number of VFSWs in HCMC as a whole.

**Results:**

Out of 24 districts, 6 were selected for the study. Mapping identified 573 venues across which 2317 unique objects were distributed in the first capture. During the recapture round, 103 venues were selected and 645 VFSWs were approached and interviewed. Of those, 570 VFSWs reported receiving the unique object during the capture round. Total estimated VFSWs in the 6 selected districts were 2616 (95% CI 2445-3014), accounting for the fact that only 25% (6/24) of total districts were selected gives an overall estimate of 10,465 (95% CI 9782-12,055) VFSWs in HCMC.

**Conclusions:**

The capture-recapture exercise provided an estimated number of VFSWs in HCMC. However, for planning HIV prevention and care service needs among all FSWs, studies are needed to assess the number of sex workers who are not venue-based, including those who use social media platforms to sell services.

## Introduction

Of the approximately 248,000 people living with HIV in Vietnam in 2017, more than 100,000 people (approximately 42%) are receiving antiretroviral therapy [1,2]. With a national estimated prevalence of about 0.3% among adults, the HIV epidemic in Vietnam remains concentrated among 3 key populations (KPs)—people who inject drugs, men who have sex with men, and female sex workers (FSWs) [2,3].

Vietnam’s capacity to further reduce HIV prevalence among the general population largely depends on knowledge and information about KPs. Population size estimates of those affected by HIV/AIDS help central and provincial level policy makers and program administrators understand the scope of the HIV epidemic, help plan appropriate interventions, allocate sufficient resources, and estimate program coverage. In Vietnam, HIV sentinel surveillance (HSS) among KPs is implemented in 20 priority provinces. The HSS provides seroprevalence and limited behavioral data. Population size estimation activity, however, has not been included in the data collection exercise [4,5]. There is limited information on FSW population size in Vietnam. In 2012, capture-recapture method was used to conduct a population size estimation activity among FSWs in Ho Chi Minh City (HCMC). The results showed that there were about 16,500 to 22,500 FSWs in the city [6]. Since then, the total population of the city had increased significantly, which may have led to a higher demand for various services. This necessitated the planning and implementation of another population size estimation activity among FSWs in HCMC. However, we decided to focus this size estimation activity among venue-based female sex workers (VFSWs) who can be reached through different venues (locations) such as restaurants, bars, massage parlors, and karaoke palaces but not all FSWs in the city for a number of reasons. First, high coverage of mobile phone users in Vietnam makes it easy for FSWs to reach their clients without being present at physical venues. Second, unavailability of practical and appropriate methods that can be used to estimate the population size of this population who approach their clients on the Internet, such as a social network platform (eg, Facebook) or messaging apps (eg, Zalo). Between December 2016 and January 2017, the Pasteur Institute in HCMC conducted the population size estimation activity among VFSWs in 6 selected districts of HCMC—the largest city in the country. The US Centers for Disease Control and Prevention and the Pasteur Institute in HCMC approved the study.

## Methods

### Overview

Before implementation, a meeting was held with FSWs and local stakeholders, including peer outreach workers, to share information about the study. Moreover, a two-sample capture-recapture method was used to estimate the population size of VFSWs in HCMC, Vietnam. VFSWs were defined as all females aged 18 years or older, having sex for money or goods in the last 3 months, residing in the city for the past 6 months, and who operate out of venues such as restaurant, coffee shops, karaoke, bars, spas, and park. Implementation of the capture-recapture method involved the following steps: selection of districts, mapping of venues, developing a list of accessible venues, distribution of unique objects to VFSWs in venues (capture), sampling of venues for recapture, and counting at the recapture sites both the number of VFSWs who received the unique object and the number of VFSWs who did not. After consulting with VFSWs and local service providers, pink makeup bags were chosen to be used as unique objects in this study (hereafter referred to as “unique objects”).

### Selection of Districts

In HCMC, there are 24 administrative districts covering approximately about 2100 km^2^, with a total population of about 9 million. In an effort to cover the city as broadly as possible, we used the HCMC Public Security Office (PSO) data and estimated the number of VFSWs that was used as an input of The Joint United Nations Programme on HIV and AIDS (UNAIDS) Estimation and Project Package (EPP) to select the districts where population size estimation activity among VFSWs was implemented.

The HCMC PSO reported that in 2013, there were about 7021 FSWs in the city disaggregated by district [7]. According to UNAIDS EPP input estimates, there are about 20,000 FSWs in HCMC. The number of FSWs reported by the HCMC PSO was used to estimate the proportion of FSWs in HCMC by district. Then, those proportions were applied to the EPP estimations to derive at an estimated number of FSWs in each district. To provide broader geographic coverage of the city, the districts were ranked from highest to lowest (column E, Table 1) and then divided into 3 groups based on the estimates. Group 1 was the top 6 districts that account for 50% of the estimated number of FSWs in the city. The second (7 districts) and third groups (11 districts) accounted for 30% and 20% of the estimated number of FSWs, respectively. To cover at least 25% of the total estimates, 6 districts were selected for the size estimation data collection activity. Moreover, 2 districts were selected at random from each group with equal probability (Table 1).

**Table 1:** District selection in Ho Chi Minh City for female sex workers.

District (A)	EPP^a^ provincial estimate distributed across districts with public security proportion (B), n	Reported FSWs^b^ by public security 2014 (C), n	Contribution of each district to EPP estimate (column B) (D), %	Cumulative (E), %	District selected (F)
Binh Thanh	3245	1139	16.22	16.22	Yes
Cu Chi	1419	498	7.09	23.32	No
District 1	1410	495	7.05	30.37	No
Go Vap	1367	480	6.84	37.20	No
Phu Nhuan	1191	418	5.95	43.16	Yes
Binh Tan	1111	390	5.55	48.71	No
Tan Phu	1014	356	5.07	53.78	No
District 10	903	317	4.52	58.30	No
Tan Binh	863	303	4.32	62.61	No
District 9	806	283	4.03	66.64	Yes
Binh Chanh	781	274	3.90	70.55	Yes
District 3	772	271	3.86	74.41	No
Hoc Mon	735	258	3.67	78.08	No
District 11	695	244	3.48	81.56	No
District 5	615	216	3.08	84.63	No
District 7	493	173	2.46	87.10	Yes
District 6	433	152	2.16	89.26	No
Nha Be	393	138	1.97	91.23	No
District 8	393	138	1.97	93.19	No
Thu Duc	345	121	1.72	94.92	No
District 12	319	112	1.60	96.51	No
District 2	251	88	1.25	97.76	No
District 4	234	82	1.17	98.93	Yes
Can Gio	214	75	1.07	100.00	No
Total	20,002	7021	100.00	—	6 districts

^a^EPP: Estimation and Project Package.

^b^FSWs: female sex workers.

### Mapping of Districts

After the districts were selected, 12 field teams—2 for each district—were formed. The field team members included 2 peer outreach workers who had prior experience of conducting outreach in their assigned districts. The field teams were then trained on method, implementation procedures, and purposes of the size estimation exercise. The training focused on how to conduct mapping including observation, documentation, maintaining confidentiality of information, and how to maintain safety in the field.

Before conducting observation and mapping, field team members were asked to generate a list of all potential venues within each of their assigned districts where they believed VFSWs congregated or worked, describe the locations, estimate the number of VFSWs that congregated in each of these venues, and the recommended times of the day and days of the week when VFSWs could be approached.

After completion of the preliminary list of venues, observation and mapping were conducted in each selected district to identify venues where VFSWs congregated or worked. During the observation, field teams also visited locations or venues within their assigned areas to ensure if any *new potential locations or venues* were added to the list. In addition, the field teams conducted brief informal *interviews* with local business owners (street-side tea and coffee shops), *xe om* (motorbike taxi operators), as well as other business establishments to corroborate the information collected based on observation and mapping.

### Development of the List of Venues

On the basis of the mapping exercise, a list of venues was created with the following information: address and description of the venues and estimated number of VFSWs congregated in each venue by days and times when they were accessible to the field team members. For the venues to be included in capture-recapture exercise, they had to meet the following criteria: (1) could be approached, (2) were safe for the study team and potential participants, and (3) had more VFSWs than the number of people who need to be approached or captured in the standardized period. It was estimated that in 1 hour, a group of 2 field team members could recruit at least 5 VFSWs. Thus, for sampling purposes, only venues that are estimated to have 5 or more VFSWs were selected to develop the list of venues. Those venues with fewer than 5 VFSWs (small venues) were combined with other venues in its proximity (about 5-min walk) and added to the list of venues. Large venues—those with 20 or more VFSWs—were divided into 2 or more venues to make sure on average these had about 5 to 10 VFSWs per venue.

### Distribution of Unique Objects (Capture)

On the basis of an initial estimate of 20,000 VFSWs and a sample size for the recapture round (554), we calculated that 2232 objects needed to be distributed to estimate the size of the population with a precision of 33% and incorporating a survey design effect of 2.0 [8]. Before distribution of unique objects, all field team members were trained on how to count potential participants, how to identify VFSWs and assess eligibility before giving them the unique objects, what to say about the unique objects, how to maintain anonymity and confidentiality of VFSWs, and how to maintain safety in the field. After the training, the field teams closely followed the schedules developed by the study team to visit all venues to distribute unique objects to all eligible VFSWs (capture exercise).

After the selection of the venues, the field team went to the venues and approached the venue owners to explain to them the purposes and procedure of the study. Once they agreed, the field team went inside the venues and approached the potential participants. The number of all potential participants during the visits was counted and recorded. Then, they were explained the purposes of the study and were asked the screening questions to determine their eligibility. If they met the selection criteria, they were informed clearly that participation was completely voluntary. The informed consent form was read to them, and verbal consents were obtained before including them in the study. Each eligible VFSW was given 1 unique object. They were also asked to keep the object because they might be asked to identify it at a later date. The object distributor used log forms to keep track of the number of unique objects distributed by date and location. No other information was collected. At the end of the distribution of unique object activity, the total number of objects actually distributed was determined. The capture exercise was completed in 2 weeks from December 5 to 18, 2016.

### Recapture

Moreover, 2 weeks after the completion of the capture exercise, a subset of venues was randomly selected with equal probability from the total list of venues used in the capture round. New field teams were formed by switching the team members within each selected district. Each new field team was assigned to approach and recruit potential participants at venues/locations where they were not assigned in the capture round. At each selected venue, the field team went to the venue and approached the venue owners to explain to them the purposes and procedure of the study. Once they agreed, the field team went inside the venues and counted and recorded all potential VFSWs. All potential VFSWs were approached, verbally consented, and assessed to make sure that they met the selection criteria. Then, they were asked whether they received any object from someone. If they mentioned that they did receive an object during the previous weeks and they could show or correctly describe and identify the unique object distributed from printed pictures showing the unique object and other 4 different objects, the VFSW was considered to be recaptured. VFSWs who mentioned that they never received any unique objects or could not describe and identify correctly the unique object were recorded as new capture in the recapture round. The recapture round was completed in 2 weeks from January 1 to 15, 2017.

### Data Analysis

Population size estimates for VFSWs across the 6 sampled districts were calculated using the formula N=MC/R, where N is the size estimate, M is the number of unique objects distributed during the capture round, C is the number of VFSWs captured in the recapture round, and R is the number of VFSWs captured on both the capture and recapture rounds [9]. Survey-weighted logit-based 95% CIs for *P* values were calculated using Taylor-linearized variance estimates to account for the selection of districts and venue, and these were then used to calculate bounds for N. The estimate for N and associated bounds were then multiplied by the inverse of the district sampling fraction to scale from the 6 sampled districts to all 24 districts in the city. The estimates for the estimated proportion of VFSWs who reported receiving an object during the recapture round and CI calculations included weights to account for district sampling probabilities and stratum and district and venue variables to account for the sampling design. The calculations were conducted in Stata version 14 (StataCorp LLC, College Station, TX).

## Results

In the capture round, a total of 2317 VFSWs received unique objects across all 573 mapped venues in the 6 selected districts. In the recapture round, 103 venues were selected and 645 VFSWs were interviewed. Of those, 88.67% (2317/2613; 95% CI 76.9% to 94.7%) were found to have received a unique object. Dividing the number of objects distributed by this estimate and bounds gives 2616 (95% CI 2445-3014) as the number of VFSWs in the 6 sampled districts. Furthermore, multiplying by 4 to account for the fact that only 6 of the 24 districts were selected gives an overall estimate of 10,465 (95% CI 9782-12,055) for the size of the VFSWs population in HCMC. Table 2 below provides the information on each of the selected districts as well as the total for HCMC.

**Table 2:** Summary of capture and recapture for venue-based female sex workers in Ho Chi Minh City.

Stratum	District	Venues identified	Unique objects distributed	Venues in recapture	VFSWs^a^ in recapture	VFSWs with unique object in recapture	Percentage of VFSWs with unique object	Estimated number of VFSWs
High	Binh Thanh	98	438	20	110	96	87.3	502
High	Phu Nhuan	62	397	14	113	88	77.9	510
Middle	Binh Chanh	174	597	28	148	135	91.2	654
Middle	District 9	45	185	9	95	77	81.1	228
Low	District 4	54	211	8	43	43	100.0	211
Low	District 7	130	489	24	136	131	96.3	508
	Total	563	2317	103	645	570	88.6^b^	2616

^a^VFSWs: venue-based female sex workers.

^b^Point estimates weighted to account for the probability of district selection and CIs calculated to account for clustering within venues.

After completing data analysis, a half-day consultation was held to present the findings to the stakeholders in the city and to discuss the challenges and limitation of the findings. The consultation was attended by officials from the HCMC Provincial AIDS Center, senior field workers, and scientific staff from the Pasteur Institute in HCMC and the US Centers for Disease Control and Prevention. The presentations included a brief description of the capture-recapture method, how it was implemented in HCMC, the results, and how to interpret the findings.

## Discussion

### Principal Findings

The data collection activity was conducted in 6 randomly selected districts to estimate the size of VFSWs aged 18 years or older. We considered 2 options for extrapolating from the 6 sampled districts to the entire city. First was to inflate the results for the 6 districts by a factor of 4 as we sampled one-fourth of the districts. The second was to inflate the size estimate for the 6 districts by the ratio of the number of females aged between 18 and 49 years in the city divided by the number in the 6 districts, based on 2016 census estimate. Using 2016 census estimates, we calculated that the proportion was 4.067 [10]. With this factor of 4.067, the estimated population size of VFSWs in HCMC would be similar to what had been estimated, accounting for the fact that only 25% (6/24) of total districts were selected (10,465).

With the advent of technology and internet, it is possible that over the years, the nature of sex work has changed. However, a significant proportion of FSWs continue to operate through different venues. In this exercise, VFSWs operated at various types of venues such as restaurants, massage parlors, bars/karaoke, cafeterias, and hostels. However, only few locations on streets where FSWs could be found were recorded. The reduction in the number of street venues might be explained by the influence of mobile phone technology, significant increase of internet users in Vietnam, and the presence of police [11]. The modern technology has helped VFSWs to reach their clients without being present at a physical location. Those who continue to operate through the venues represent a segment of the FSWs’ population in HCMC who are somewhat visible and accessible to health care workers. Significant efforts were made to cover all venues where sex workers could be approached during capture and recapture; however, the field staff reported that because of many factors (approachability and security issues), only about 70% of venues in the selected districts during mapping were covered. If the missed venues were similar to those included in the study, then the VFSW population size for HCMC would be estimated at 10,465/0.70 or 14,950. This estimated size of the population is lower than that of a study using a capture-recapture method (19,602; 95% CI 16,590-22,554) but is higher than high the estimate of VFSW in the same study using data from police system (10,595) in 2013 [12,13].

### Limitations

There are a number of limitations to this study. First, the findings presented above are limited to those VFSWs in HCMC who are accessible only at selected venues. Findings do not include VFSWs who operate out of venues that were not identified and not included in the list of venues for data collection. In a large city such as HCMC, there are venues the study teams were not able to access, such as *high-class* restaurants, massage parlors, or entertainment venues. Other FSWs who do not operate out of venues were not accessed and could not be included in the estimation either. Second, the estimation does not include FSWs who connect to their client through modern technology such as Web or telephones. Recent figures show that Vietnam has the largest and fastest growing smartphone and internet users in the world, with an estimate of over 45% of the population using smartphones and internet [11]. Therefore, it is possible FSWs are using other methods such as telephones and internet to connect with prospective clients. Third, in Vietnam, sex work is illegal; the public security authorities maintain a relatively close watch on individuals who may be working as sex workers, making the hidden populations even more difficult to reach. That primarily limits the street operations of FSW. However, this may also limit those who operate at various venues. In addition to public scrutiny, the venue owners control the number of FSWs at any venue at a given time. They control their working hours and how and when they can be accessed. An illustration of this was a very low number of VFSWs venues existed during the mapping, and not all venues could be accessed. Police activity in some of the districts may have influenced the number of VFSWs who could be approached during the size estimation exercise. Fourth, as we recruited those aged 18 years or older, the population size estimates of VFSWs did not include those aged younger than 18 years. Finally, the assumption of *closed population* might have been violated. However, it was minimized by shortening the time of the study (in about 6 weeks) and the time between the 2 samples (2 weeks) and avoiding to collect data on unusual days (such as Lunar New Year). Despite the potential limitations, the findings of this study are useful for program planning and assessments that highlight the importance of designing appropriate interventions for VFSWs in HCMC. The findings also highlight the important need to investigate possible ways to estimate those who do not operate out of venues, especially those who use various social media platforms; their characteristics; and how they could be reached with appropriate interventions.

## Abbreviations

EPP: Estimation and Project Package
FSW: female sex worker
HCMC: Ho Chi Minh City
HSS: HIV sentinel surveillance
KP: key population
PSO: Public Security Office
UNAIDS: United Nations Programme on HIV and AIDS
VFSW: venue-based female sex worker
